# Twist and chew: three-dimensional tongue kinematics during chewing in macaque primates

**DOI:** 10.1098/rsbl.2021.0431

**Published:** 2021-12-15

**Authors:** Kara L. Feilich, J. D. Laurence-Chasen, Courtney Orsbon, Nicholas J. Gidmark, Callum F. Ross

**Affiliations:** ^1^ Department of Organismal Biology and Anatomy, University of Chicago, Chicago, IL, USA; ^2^ Department of Radiology, University of Vermont Medical Center, Burlington, VT, USA; ^3^ Biology Department, Knox College, Galesburg, IL, USA

**Keywords:** feeding, biomechanics, X-ray reconstruction of moving morphology

## Abstract

Three-dimensional (3D) tongue movements are central to performance of feeding functions by mammals and other tetrapods, but 3D tongue kinematics during feeding are poorly understood. Tongue kinematics were recorded during grape chewing by macaque primates using biplanar videoradiography. Complex shape changes in the tongue during chewing are dominated by a combination of flexion in the tongue's sagittal planes and roll about its long axis. As hypothesized for humans, in macaques during tongue retraction, the middle (molar region) of the tongue rolls to the chewing (working) side simultaneous with sagittal flexion, while the tongue tip flexes to the other (balancing) side. Twisting and flexion reach their maxima early in the fast close phase of chewing cycles, positioning the food bolus between the approaching teeth prior to the power stroke. Although 3D tongue kinematics undoubtedly vary with food type, the mechanical role of this movement—placing the food bolus on the post-canine teeth for breakdown—is likely to be a powerful constraint on tongue kinematics during this phase of the chewing cycle. The muscular drivers of these movements are likely to include a combination of intrinsic and extrinsic tongue muscles.

## 1. Background

**Figure 1.**
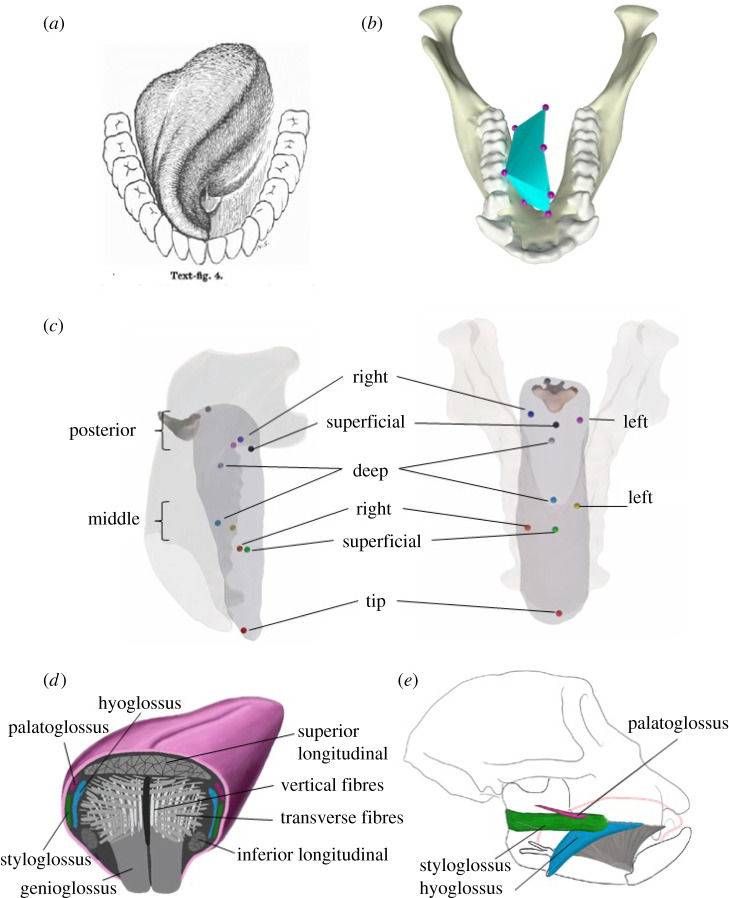
(*a*) Abd-el-Malek's sketch of human tongue shape during chewing on the right side tooth row [5]. (*b*) Still frame of macaque tongue in similar position. (*c*) Marker locations in animal J. (*d*) Cross section of tongue showing extrinsic and intrinsic musculature. Modified from Orsbon *et al.* [6]. (*e*) Location of extrinsic tongue muscles in the macaque. Modified from Orsbon *et al.* [6].

Mammal tongues can assume a wide range of shapes during vocalization, grooming and feeding. The tongue is an especially important actor in feeding, sensing the location and properties of the food bolus, positioning it between the teeth during chewing and propelling it into the pharynx during swallowing. In primates and other mammals that often chew on only one side at a time (the biting or ‘working side’), complex asymmetrical tongue movements are essential for positioning the food bolus between the teeth, so it can be fractured in preparation for swallowing. Because the tongue is largely hidden within the mouth, it has been difficult to make detailed measurements of these movements and shape changes during feeding [1,2] and to relate these tongue movements to simultaneous mandible movements. Such information is important context for studies of tongue movement during feeding, as rehabilitation of tongue movements, if and where possible, must occur in coordination with the mandible for feeding to be successful. Abd-el-Malek [3] used photography to suggest that during chewing by humans, the middle of the tongue twists to the biting side to position the food bolus between the teeth as they come together. Although Abd-el-Malek's model of tongue kinematics during chewing is often referred to and has received some support over time [4], it is purely qualitative (consisting of sketches, e.g. figure 1*a*), it has not been quantitatively tested using three-dimensional (3D) tongue kinematic data and his descriptions of tongue kinematics are difficult to relate to simultaneous mandible kinematics. His sketches also suggest that twisting of the dorsum of the middle tongue to the working side is accompanied by twisting of the tongue tip towards the balancing side (figure 1*a*). Abd-el-Malek does not comment on this phenomenon, but it is described in hemiplegic monkeys [7] and may reveal insight into mechanisms underlying tongue movement and control.

Videofluoroscopic techniques deployed to study tongue movements in humans and other primates have been predominantly two-dimensional (2D) and in lateral view. Consequently, although anteroposterior (AP) and superoinferior tongue kinematics in sagittal planes are well documented [5,8,9], the difficulty of resolving tongue movements in frontal view means that the few published studies of mediolateral tongue movements are largely qualitative [4,10]. One study inferred twisting of the tongue during chewing from 2D cinematographic data on the movement of lead markers glued to the tongue surface in asynchronous lateral and frontal views [11]. Without collection of radiographic data in two views simultaneously, it is impossible to make precise and accurate measures of tongue movements in 3D, leaving significant gaps in our understanding of how tongue and mandible movements are coordinated and controlled by the central nervous system.

The recent development and dissemination of the X-ray reconstruction of moving morphology (XROMM) workflow for analysis and visualization of biplanar videoradiographic data [12,13] makes it possible to quantify 3D tongue shape and position using small spherical tantalum bead implants [6,14–16]. To date, detailed descriptions of high-resolution 3D tongue kinematics have been limited to measures of tongue protraction, retraction, width and length during chewing and drinking in pigs [14,15,17]; data on tongue flexion and roll, and tongue kinematics during chewing in a non-human primate model of human feeding have not been presented. Here, we report XROMM-based quantification of 3D tongue kinematics during mastication in macaque primates and document their relationships to simultaneous mandible movements. We hypothesized that during mandible depression and early mandible elevation of chewing gape cycles, the middle of the tongue's dorsal surface twists to the working side through a combination of roll about the tongue's long axis and flexion in sagittal planes, accompanied by twisting or bending of the anterior tongue back towards the balancing side [3,4,11] (figure 1*a*).

## 2. Material and methods

### (a) Animal subjects

All procedures were approved by the University of Chicago IACUC. Four adult rhesus macaques (*Macaca mulatta*) were observed in this study (table 1). The animals were housed in an AAALAC-accredited animal facility, fed monkey biscuits and given daily enrichment. They were trained to feed while restrained in a radiolucent, acrylic primate chair. Monkeys C and H were trained to feed with their heads restrained, facilitating marker tracking, shortening recording durations and reducing radiation exposure. The monkeys were fed red grapes (10–20 mm), and biplanar videoradiographic data were collected (technique: 90–100 kVp, 10–16 mA, 200 Hz and with a 2–4 ms shutter speed). Swallow cycles, identified following [8], were excluded.

**Table 1.** Animal sex, age, mass and differences in timing between kinematic extremes with 99% confidence intervals.

peak events being compared	animal C	animal H	animal J	animal K
	(male, 9 years, 8.8 kg)	(female, 8 years, 7.5 kg)	(male, 16 years, 8.5 kg)	(female, 12 years, 7.5 kg)
sagittal flexion versus middle tongue roll	−8.6 ± 2.8%^a^	−11.1 ± 5.0%^a^	−6.4 ± 3.9%^a^	−10.6 ± 4.8%^a^
balancing anterior strain versus sagittal flexion	8.1 ± 2.3%^a^	6.1 ± 5.4%^a^	3.6 ± 4.2%	5.8 ± 3.0%^a^

^a^Indicates significantly different from 0. A negative number indicates the first event listed occurs before the second event listed.

3D rigid body kinematics of cranium, mandible and hyoid, as well as 3D movements of 1 mm tantalum markers in the tongue, were quantified using biplanar videoradiography and the XROMM workflow [6,16]. Tantalum beads were implanted in the cranium, mandible and hyoid through small holes drilled in cortical bone. Nine markers were implanted in the tongue through hypodermic needles: one near the tongue tip, four posterior markers roughly in a coronal plane at the level of the palatoglossal arch (posterior tongue plane) and four middle markers roughly in a coronal plane halfway between the posterior markers and the tip (middle tongue plane). The tongue tip marker was implanted just deep to the mucosa; the four middle and four posterior markers included two lateral markers under the mucosa on the sides of the tongue, a superficial midline marker under the mucosa and a deep midline marker *ca* 10 mm deep (figure 1*c*). The non-rigidity of the tongue resulted in some inter-individual variation in marker placement (electronic supplementary material, figure S1).

A cranial coordinate system was established from the XYZ coordinates of the landmarks on 3D bone models reconstructed from CT scans, and the following analyses performed using custom code in R (R v. 4.0.3, [18]). Mandibular pitch was quantified using a joint coordinate system [19] following conventions in [16]. Chewing gape cycles were defined from minimum gape to minimum gape, and the four vertical kinematic phases of the gape cycle were identified using mandibular pitch angle [20]. Slow open (SO) phase starts at minimum gape and ends as mandible depression velocity increases into fast open (FO). Maximum gape marks the end of FO and the start of fast close (FC), during which the mandible elevates rapidly. As the teeth contact the food, mandible velocity decreases, marking the start of slow close (SC), which ends at minimum gape. The direction of mandible yaw during SC was used to identify chewing side.

In the anatomical position with the mandible elevated and the tongue at rest, the tongue tip is anterior, the palatal surface of the oral tongue is superior, there are left and right sides and the tongue's long axis is in the mid-sagittal plane. The superior (palatal) surface of the oral tongue is the dorsum, so that inferior bending in a plane perpendicular to the superior surface—decreases in inferior angles along the tongue's long axis—is sagittal flexion. Lateral flexion occurs in planes parallel to the superior surface, and right and left roll describe rotation of the dorsum about the tongue's long axis, towards the given side.

**Figure 2.**
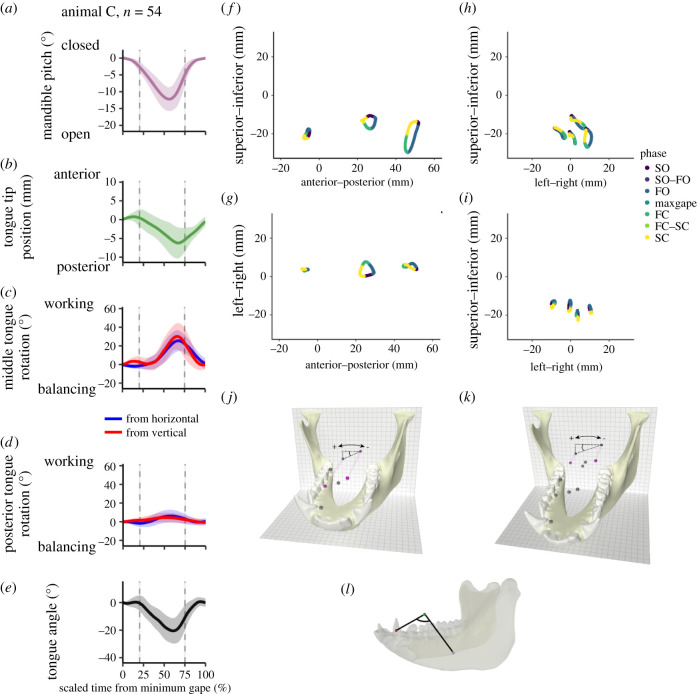
Tongue and mandible kinematics during right chewing gape cycles in animal C. Gape cycles are standardized in length: ordinate is the proportion of total gape cycle duration. Data are means ± s.e.m. (*a*) Mean mandible pitch angle (in degrees). Gape cycles begin and end at minimum gape (mandible elevated). Vertical dashed lines in all graphs indicate mean timing of SO/FO transition and of FC/SC transition. FO ends and FC begins at maximum gape (maximum mandible depressed) (mean timing not shown). (*b*) Tongue tip AP position (in mm) in cranial coordinate system relative to reference position at minimum gape. (*c*) Roll of middle tongue (degrees) using vertical and horizontal axes, relative to reference position at minimum gape. (*d*) Roll of posterior tongue (degrees) using vertical and horizontal axes, relative to reference position at minimum gape. (*e*) Tongue axis flexion angle relative to angle at minimum gape. (*f*) Lateral view of midline markers over an average chew cycle. Positive on the *x*-axis is anterior, on the *y*-axis is superior. Colour indicates cycle phase. (*g*) Axial (dorsal) view of midline tongue markers over an average chew cycle. Positive on the *x*-axis is anterior, on the *y*-axis is left. (*h*) Coronal view of middle tongue markers over an average chew cycle. Positive on the *x*-axis is left, on the *y*-axis is superior. Colour indicates cycle phase. (*i*) Coronal view of posterior tongue markers over an average chew cycle. Positive on the *x*-axis is left, on the *y*-axis is superior. Colour indicates cycle phase. (*j*) Example measurement of middle tongue horizontal rotation. (*k*) Example measurement of posterior tongue horizontal rotation. (*l*) Example measurement of sagittal tongue flexion angle. Note that plot (*e*) shows this measurement relative to the same measurement made at rest.

Tongue kinematics were quantified in the cranial coordinate system using orientations and lengths of lines between the tongue markers (figure 2*j–l*). Roll was quantified using rotations of vertical and horizontal lines in middle and posterior coronal planes through the tongue. Vertical lines connect superficial midline markers with either the hyoid (posterior) or deep midline (middle) markers; horizontal lines connect lateral markers in middle and posterior tongue planes; positive roll is anticlockwise looking at the tongue from the front. Lateral flexion was quantified using strain (% change) in Euclidean distances between lateral markers in middle and posterior planes (posterior tongue) and between lateral middle markers and the tongue tip marker (anterior tongue). Working side flexion is contraction of inter-marker distances on the working side; balancing side flexion is the reverse. Tongue sagittal flexion angle was calculated from the anterior, middle superficial and middle hyoid landmarks using the dot product and the law of cosines. Each of these metrics was compared on a timescale from minimum gape to minimum gape: 0% is the starting minimum gape, and 100% is the following minimum gape—capturing the main tongue kinematic events bracketing maximum gape. All distances and angles were normalized to distances at rest (minimum gape).

To compare the relative timing of kinematic events, we calculated the 99.5% confidence interval of the mean paired difference between scaled time of the two events [21]. If the confidence interval excludes zero, then there is a statistically significant difference between the timings. Scaled times were rescaled to a 0–360° domain to calculate statistics and then converted back to 0–100% for interpretation. An alpha value of 0.005 was chosen to be conservative in the face of multiple testing. Box plots of timing of events are given in electronic supplementary material, figure S3.

## 3. Results

Sagittal plane tongue movements during chewing cycles in macaques are well known [5,8]: at minimum gape, as the mandible starts to slowly open, the tongue tip is protracting (figure 2*b*; electronic supplementary material, figure S2B). Around the end of SO and the start of FO, the tongue tip starts to retract, and it continues retracting throughout FO and much of FC, reversing the direction to start protracting again around the start of SC. This phase of anterior tongue retraction, coincident with the fast phases of the gape cycle, is when the majority of tongue twisting and bending occurs.

At the start of FO, the middle tongue plane starts to roll towards the working side, and the long axis of the tongue starts to flex (i.e. bend inferiorly) (figure 2*c*,*e*) (electronic supplementary material, online video). Both these movements peak (with rotations of *ca* 20–30°) during FC, but sagittal flexion peaks first (table 1). Also starting with FO, the distance between the middle tongue plane and the tip contracts on the balancing side (electronic supplementary material, figure S2E). In two animals (C, H) the working side tongue tip distance does not decrease, so this balancing side contraction indicates balancing side flexion of the tongue tip. In J and K, both working and balancing side distances initially decrease together—anterior tongue shortening—but even in these animals, balancing side anterior tongue contraction persists after the working side starts to lengthen: in all but J, the tip of the tongue flexes to the balancing side after sagittal flexion has peaked (table 1).

Rotations of the posterior tongue plane are similar in sign but are much smaller in magnitude (less than 23–24°) than those of the middle plane. They have only a small role in tongue deformation during chewing. Posterior tongue strains—between middle and posterior planes—also seem to play a minor role in tongue shape change during chewing: the magnitudes of the strains are low, and they do not peak in synchrony with either middle tongue rotations or long axis flexion.

## 4. Discussion

Macaque tongue kinematics at the end of FC resemble Abd-el-Malek's drawings of human tongue shapes during chewing; the middle of the tongue is rolled over towards the working side, and the tongue tip is yawed back to the balancing side. The timing of this posture—just prior to the FC/SC transition—confirms Abd-el-Malek's hypothesis that this movement positions the food bolus between the working side cheek teeth during chewing in macaques and humans [3,4]. Posterior tongue roll and yaw play little role in overall tongue rotation during chewing in macaques, presumably because the back of the tongue is anchored to the hyoid bone, palatoglossus, styloglossus and hyoglossus. Middle tongue roll and long axis flexion also appear to occur during chewing in pigs and may explain regional changes in tongue lengths and widths [14,15].

How is bending and twisting of the tongue produced? Abd-el-Malek suggested that this posture is due to contraction of the balancing side styloglossus muscle, running from the styloid process into the side of the posterior tongue. Others suggest that tongue rotation is produced by contraction of working side hyoglossus, styloglossus and longitudinal intrinsic muscles, along with balancing side styloglossus and transverse intrinsic muscles [11]. The styloglossus of *M. mulatta* is more horizontally oriented than that of humans, but it also inserts into the side of the back of the tongue with its force vector posteriorly oriented ([16]: figure 1). Unilateral contraction of styloglossus and transmission of its force through the tongue tissues could plausibly flex the tongue's long axis and rotate the middle of the tongue to the other side; whether it could produce the simultaneous balancing side tongue tip flexion is unclear. Even if this ‘styloglossus hypothesis' is correct, the subtle dynamics of the relative timing of our measures of tongue deformation suggests that contraction of a single muscle is unlikely to be the only mechanism deforming the tongue during the FO and FC phases of chewing [1]. As in humans, the core of the macaque tongue is formed by short, transversely and vertically oriented intrinsic muscle fibres interleaved with the superior terminations of the genioglossus muscle (figure 1). This core is surrounded by a superior band of intrinsic longitudinal muscles along the top of the tongue, two inferior bands of intrinsic longitudinal muscles inferiorly and longitudinally oriented terminations of the extrinsic styloglossus, hyoglossus and palatoglossus muscles flanking the core of the tongue posteriorly [16]. The tongue of therian mammals appears to act as a constant volume structure overall, so unilateral contraction of longitudinal intrinsic muscles is one plausible mechanism contributing to the flexion and twisting documented here. Such contraction could result in deformation, followed by torsion to keep the tongue isovolumetric. However, more data on the activation of longitudinal intrinsic muscles are necessary to test this hypothesis.

## 5. Conclusion

This is the first quantification of 3D twisting and flexion of the tongue during chewing: the tongue posture illustrated in sketches by Ab-el-Malek is shown to occur during the FO and FC phases of the gape cycle and to be produced by sagittal flexion and middle tongue roll, accompanied by balancing side flexion of the tongue tip. Despite the behavioural flexibility of the tongue during vocalization, drinking and food ingestion, the movements during chewing seem remarkably stereotyped: they were very similar in all four animals (eating grapes) and resemble those illustrated for humans eating a wide range of foods, including nuts, candies and gum. Although 3D tongue movements during chewing will undoubtedly vary with food type to some degree, if the primary function of working side roll of the middle tongue is to position a food bolus between the teeth for trituration, it is likely that food effects might manifest in other dimensions of tongue movement. Qualitative observations of tongue kinematics in pigs during chewing suggest twisting and flexion may also occur in other mammals [4]. The neuromuscular mechanisms underlying these movements are as yet unknown, but unravelling them will be important for understanding tongue evolution in mammals, as well for treatment of neuromuscular pathologies of the tongue affecting feeding and vocalization. The strong resemblance between tongue posture in hemiplegic monkeys [7] and that observed during chewing here suggests that insight into the muscular control of tongue movement during chewing may inform treatments for muscular and neural control deficits following stroke.

## Data accessibility

All data and code used to produce the results and figures in this manuscript, as well as a readme file describing their use, are available in a Dryad Digital Repository: https://doi.org/10.5061/dryad.bk3j9kdd2 [22].
